# The role of tyrosine kinase inhibitors as a post-hematopoietic stem cell transplant continuation therapy for pediatric acute leukemia: a systematic review

**DOI:** 10.1007/s00280-026-04976-9

**Published:** 2026-10-27

**Authors:** Mahati Avineni, Yara K. Habo, Nora K. Habo, Ahmed Adham R. Elsayed, Marc D. Basson

**Affiliations:** 1 College of Graduate Studies, Northeast Ohio Medical University, Rootstown, OH 44272 USA; 2 College of Medicine, Northeast Ohio Medical University, 4209 St, OH-44, Rootstown, OH 44272 USA; 3 Department of Surgery, Northeast Ohio Medical University, 4209 St, OH-44, Rootstown, OH 44272 USA; 4 Department of Biomedical Sciences, Northeast Ohio Medical University, 4209 St, OH-44, Rootstown, OH 44272 USA

**Keywords:** Leukemia, Hematopoietic stem cell transplantation, Pediatric, Continuation therapy, Tyrosine kinase inhibitors

## Abstract

**Purpose:**

Relapse following hematopoietic stem cell transplantation (HSCT) is a leading cause of transplant failure for pediatric acute leukemia patients, emphasizing the need for an effective post-transplant continuation therapy. This systematic review evaluates the role of tyrosine kinase inhibitors (TKIs) as a post-HSCT continuation therapy in pediatric acute leukemia.

**Methods:**

A literature search of PubMed, Cochrane, Web of Science, and VHL was conducted according to PRISMA guidelines. Original studies were included if they were relevant to the research question and fit the eligibility criteria.

**Results:**

The initial search identified 765 articles, with 16 studies included after filtration. TKIs were indicated for patients with chromosomal alterations causing constitutively active kinase signaling. Common agents included imatinib, dasatinib, ponatinib, sorafenib, and gilteritinib, and selection was based on disease status and molecular profiling. Duration of continuation ranged from 10 months to 3 years, with dosage dependent on the agent and tolerability. TKIs were generally well-tolerated, with reported side effects including cytopenia, liver dysfunction, and skin conditions. Across the included studies, MRD negativity and sustained molecular remission were reported in selected patients receiving post-HSCT TKI continuation therapy, while relapse was also reported after treatment discontinuation or in resistant disease. However, optimal duration and long-term safety remain unclear in the pediatric population.

**Conclusion:**

TKIs can serve as a targeted post-HSCT continuation therapy in select pediatric acute leukemia patients, particularly those with high-risk, mutation-driven disease. Molecular profiling and MRD monitoring are central to guiding agent selection, timing, and mutation-directed therapy adjustments. These findings support individualized TKI treatment to reduce relapse risk following HSCT, while highlighting the need for prospective studies to define standardized treatment strategies.

**Graphic abstract:**

**Figure:**
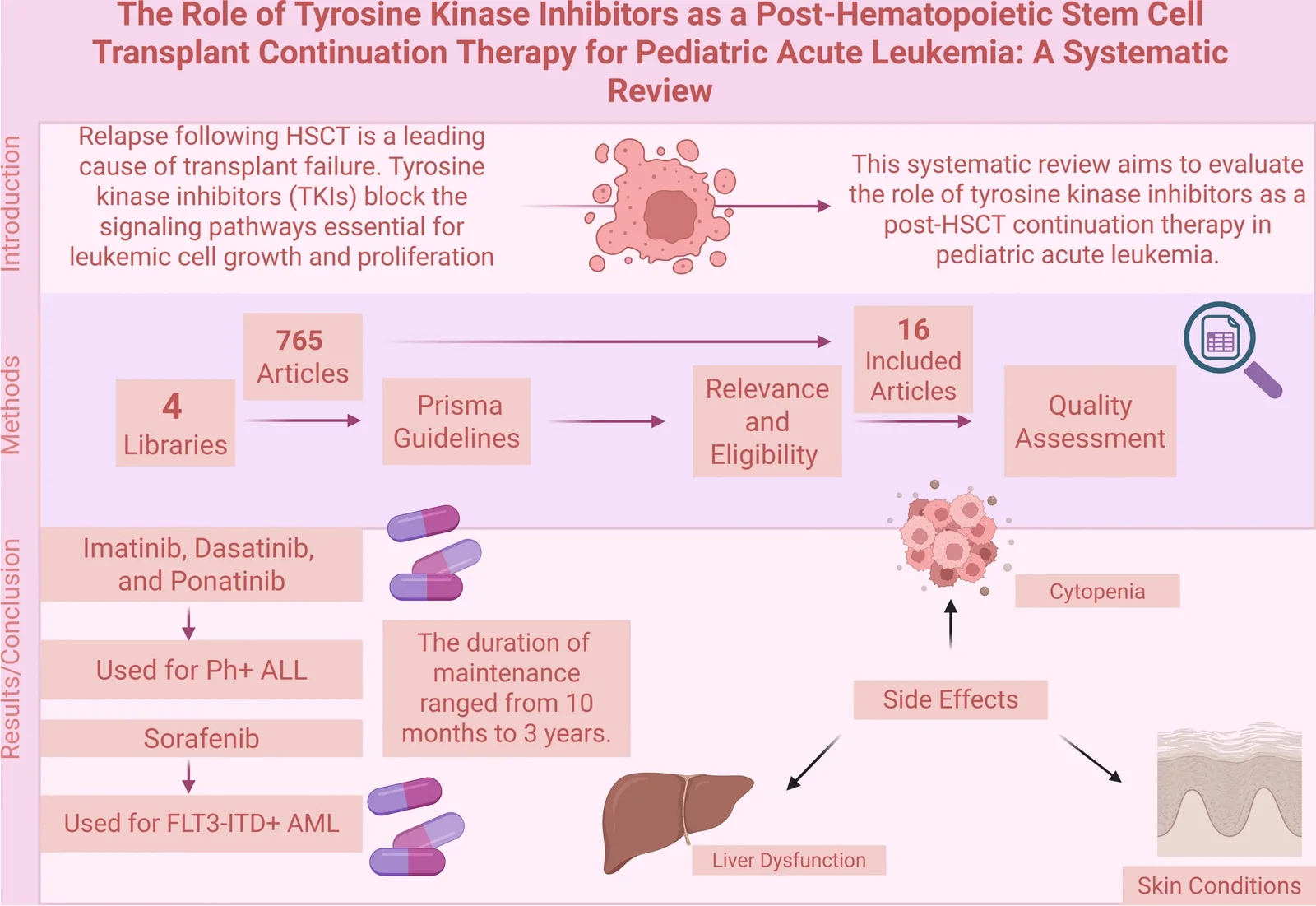

**Supplementary Information:**

The online version contains supplementary material available at https://doi.org/10.1007/s00280-026-04976-9.

## Highlights

Post-HSCT TKI continuation can reduce relapse risk in pediatric acute leukemia.

Agent selection is guided by disease status and molecular profiling.

Ongoing MRD surveillance and individualized continuation strategies are necessary.

Pediatric dosing and continuation duration remain undefined.

**Supplementary Information:**

The online version contains supplementary material available at https://doi.org/10.1007/s00280-026-04976-9.

## Introduction

Hematopoietic stem cell transplantation is a critical component of curative therapy for pediatric acute leukemia patients and those classified as high risk or patients who relapse following standard treatment [1]. Autologous and allogeneic stem cell transplants are both used in pediatric acute leukemia. However, their clinical roles differ [2]. Autologous HSCT avoids complications of graft-versus-host disease, but it lacks the graft-versus-leukemia effect that potentiates treatment and is therefore associated with higher relapse rates [2]. Allogeneic HSCT is preferred for high-risk/relapsed patients due to its graft-versus-host-leukemia effect, despite the increased risk of infection, organ toxicity, and GVHD [2]. Proper donor selection is critical in the treatment of patients receiving HSCT. Selection is determined based on HLA compatibility, donor availability, disease status, and treatment urgency [3]. Despite adequate donor matching and pre-transplant treatments, relapse following HSCT remains a leading cause of transplant failure, emphasizing the importance of effective post-transplant continuation treatments [4]. This systematic review aims to evaluate the role of tyrosine kinase inhibitors as a post-transplant continuation therapy in pediatric acute leukemia.

Tyrosine kinase inhibitors are targeted therapies that inhibit signal transduction pathways essential to leukemic cell proliferation and survival [5]. TKIs are widely associated with the treatment of many cancers and are the first-line treatment of Philadelphia chromosome–positive acute lymphoblastic leukemia (Ph+ ALL) and other molecular subtypes of leukemia [5]. After transplantation, TKIs offer a continuation approach by targeting residual disease and enhancing graft-versus-leukemia effects while avoiding the toxicity associated with additional intensive chemotherapy [6].

Although they are frequently used in this setting, the role of TKIs as post-transplant continuation therapy in pediatric acute leukemia remains incompletely defined. Variability in leukemia subtype, molecular targets, timing and duration of therapy, and toxicity profiles limits the development of standardized clinical guidelines. Relapse following HSCT is a continuous clinical challenge in the treatment of acute leukemia [4]. This systematic review aims to synthesize the current literature to inform clinical decision-making and guide future research.

## Methods

### Search strategy

This systematic review was conducted according to PRISMA guidelines. The search was conducted on October 12th, 2025, across four databases, including PubMed, Cochrane, Web of Science, and VHL. The main keywords used were “leukemia”, “hematopoietic stem cell transplantation”, “pediatric”, “maintenance therapy”, and “tyrosine kinase inhibitors”. The appropriate synonyms and MeSH terms were included when available. (See Supplementary Table 1 for the full query.) Two independent reviewers filtered through the articles by title and abstract, followed by a full-text review. Articles were filtered based on the eligibility criteria and relevance to the following research question: “What is the role of tyrosine kinase inhibitors as a post-HSCT continuation therapy for pediatric acute leukemia?” Any disagreements between the two reviewers were resolved by consensus. If consensus could not be reached, senior authors were consulted. The review was not prospectively registered in a systematic review registry.

### Eligibility criteria

Articles were excluded if they were not written in English, did not include human subjects, or were non-original studies such as literature reviews, meta-analyses, or systematic reviews. Studies were included if patients were pediatric (ages 0 to 18) with pediatric results reported separately from any adult patient results. Additionally, included studies had patients diagnosed with acute leukemia who underwent a hematopoietic stem cell transplant and were treated with a tyrosine kinase inhibitor post-transplant.

### Data extraction

After filtration, all included studies were exported to Zotero. Demographic and characteristic data were extracted for the following information: author and year published, study design, total population, relevant population, age, sex, and population inclusion criteria. The summary of outcomes (Supplementary Table 2) was also extracted based on the following factors: type of leukemia, type of TKI, dosage and duration, indication and practical consideration in pediatrics, biological mechanism and clinical foundation, effect of genetic mutation, treatment side effects and related toxicity, impact on transplant-related outcome, minimal residual disease or prophylactic usage, clinical implications, and mechanism of resistance.

### Quality assessment and risk of bias

Quality assessment for each study was done according to the EQUATOR guideline based on the study design. Observational studies were quality assessed using STROBE, and case reports and series were quality assessed using CARE. Risk of bias for the observational studies was assessed using ROBINS-I.

## Results

### Search results

**Fig. 1:**
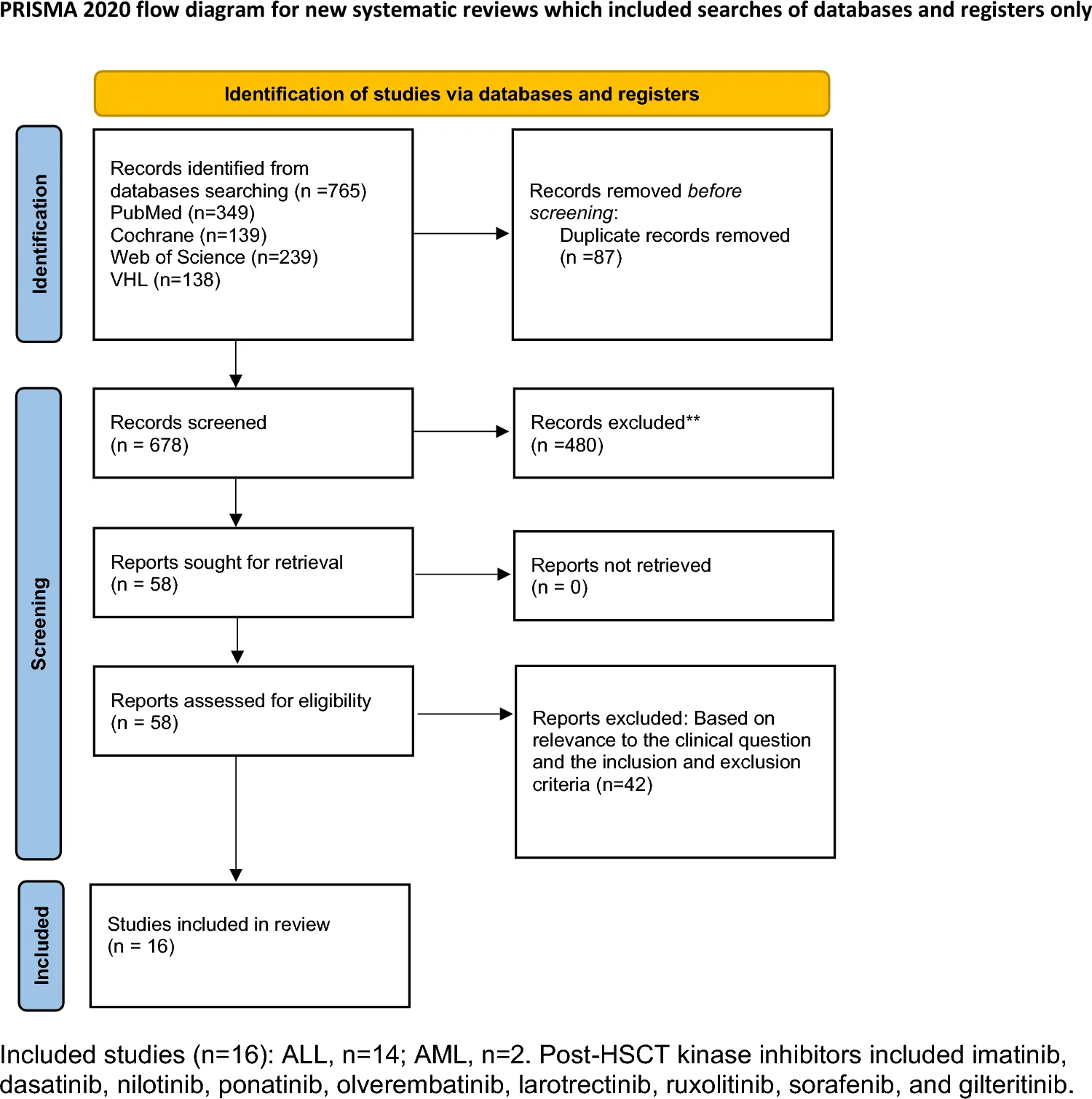
PRISMA flow diagram for systematic reviews, including searches of databases and registers

The initial search yielded a total of 765 articles. After duplicates had been removed, the remaining number was 678. The title and abstract review, followed by the full text review, narrowed this down to 16 final studies included in this systematic review (Fig. 1).

### Demographics and characteristics

Of the 16 included studies, the oldest was published in 2004, and the most recent in 2025 [7, 8]. The total population described was 118, and the relevant population was 94. The study with the highest relevant population included 44 patients, and the lowest described 1 patient [9, 10]. The oldest included patient was 18 years old, and the youngest was 3 years old [9, 11]. All included studies consisted of pediatric patients diagnosed with acute leukemia who underwent HSCT and were treated with a TKI post-transplant (Table 1).

**Table 1:** Demographics and Characteristics

Author, year	Study design	Total population	Relevant population	Age (Mean ± SD or Range)	Sex	Leukemia subtype	Post-HSCT TKI(s) used	Population inclusion criteria
Byun et al., [21]	Retrospective Cohort Study	15	7	3.1–15.8 years	Male: 10 Female: 5	ALL	Imatinib	Pediatric Ph+ ALL, confirmed t(9;22)/BCR-ABL, treated at center, completed induction, eligible for HSCT
Chen et al., [9]	Retrospective Cohort Study	50	44	4–18 years	-	ALL	Imatinib, ponatinib	-Had Philadelphia chromosome–positive -Received haploidentical allogeneic HSCT -Were planned to receive or eligible for post-transplant imatinib prophylaxis -Had measurable BCR-ABL transcripts for MRD monitoring
Chen et al., [22]	Prospective Cohort Study	26	6	5–16 years	-	ALL	Ponatinib	Ph+ ALL w/ T315I; allo-HCT; eligible for ponatinib prophylactic/preemptive therapy
Dai et al., [10]	Case Report	1	1	6 years old	Male: 1	ALL	Dasatinib	-Pediatric patient (age 6) -Relapsed B-cell ALL -Underwent haploidentical HSCT -Received post-transplant dasatinib maintenance
Grossman et al., [7]	Case Report	1	1	8 years old	Male:1	ALL	Imatinib	-Pediatric age (8 years) -Diagnosis of Ph+ ALL -Received umbilical cord blood HSCT -Received imatinib post-transplant
Li et al., [12]	Retrospective Case Series	7	3	9–13 years	Male: 2 Female: 1	ALL	Olverembatinib	-Pediatric age (< 18 years) -Diagnosis of relapsed or refractory ALL -either Ph+ ALL or ABL-class fusion ALL
Lockhart et al., [13]	Case report	1	1	4	female	ALL	Imatinib, dasatinib	-pediatric age (4) -Ph+ ALL -allogenic HSCT -imatinib and dasatinib post-transplant
Millot et al., [14]	Case report	1	1	4	male	ALL	Dasatinib	-pediatric age (4) -pre B-cell Ph-positive ALL -underwent allogenic HSCT -dasatinib post-transplant
Reinwald et al., [15]	Retrospective case series	4	1	15	-	ALL	Nilotinib	-pediatric age (15) -BCR-ABL+ ALL -underwent allogenic HSCT -nilotinib for CNS relapse post-transplant
Roldán et al., [8]	Case report	1	1	15	male	AML	Sorafenib	-pediatric age (15) -FLT3-ITD+ AML -allo-HSCT -sorafenib post-transplant
Sakurai et al., [16]	Case report	1	1	9	female	ALL	Imatinib	-pediatric age (9) -BCP-ALL -cord blood transplant -imatinib pre and post-transplant
Schewe et al., [17]	Case report	1	1	6	male	ALL	Larotrectinib	-pediatric age (6) -B-cell ALL -allogenic HSCT -larotrectinib post-transplant
Shumock et al., [11]	Case series	5	3	patient 3: 7 patient 4: 14 patient 5: 3	patient 3: male patient 4: female patient 5: male	ALL	Dasatinib, ruxolitinib, and ponatinib	-pediatric age (3–14) -Ph-like B-AL -PBSCT -pre and post-transplant TKIs
Tachibana et al., [18]	Case series	2	1	15	male	ALL	Ponatinib	-pediatric age (15) -Ph+ ALL -allogenic HSCT -pre and post-transplant TKIs
Tasaka et al., [19]	Case report	1	1	15	male	AML	Gilteritinib	-pediatric age (15) -FLT3-ITD+ AML -allogenic HSCT -post-transplant TKIs
Watanabe et al., [20]	Case report	1	1	10	male	ALL	Dasatinib	-pediatric age (10) -Ph+ ALL -allogenic HSCT -pre and post-transplant TKIs

### Quality assessment and risk of bias

All 13 case reports and series were quality assessed using CARE (Table 2) [7, 8, 10–20]. All 3 observation studies were quality assessed using STROBE (Supplementary Table 3), and risk of bias was assessed using ROBINS-1 (Supplementary Table 4) [9, 21, 22].

**Table 2:**
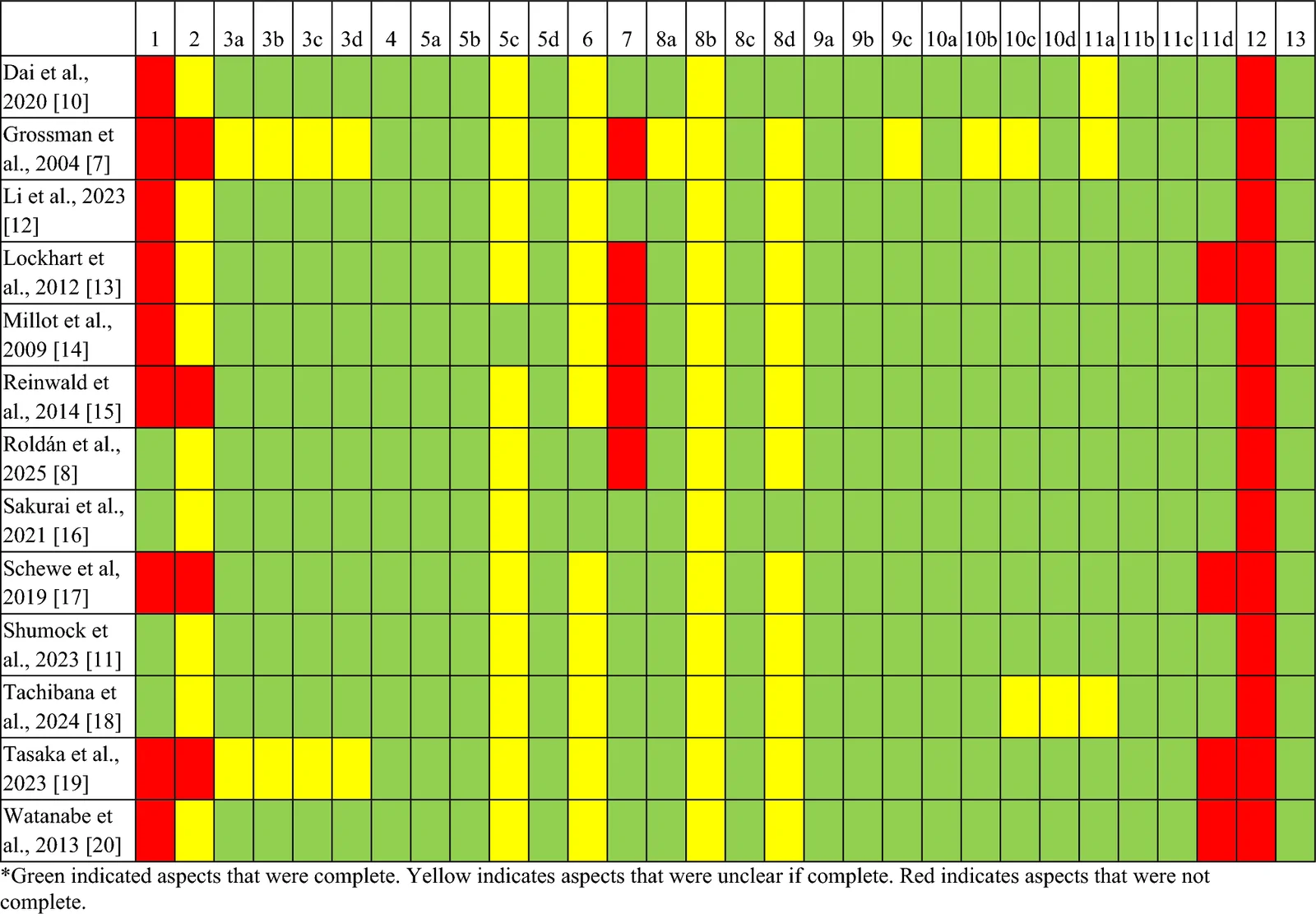
Quality assessment – CARE

1. The diagnosis or intervention of primary focus followed by the words “case report”

2. 2 to 5 key words that identify diagnoses or interventions in this case report, including "case report"

3a. Introduction: What is unique about this case and what does it add to the scientific literature?

3b. Main symptoms and/or important clinical findings

3c. The main diagnoses, therapeutic interventions, and outcomes

3d. Conclusion—What is the main “take-away” lesson(s) from this case?

4. One or two paragraphs summarizing why this case is unique (may include references)

5a. De-identified patient specific information

5b. Primary concerns and symptoms of the patient

5c. Medical, family, and psycho-social history including relevant genetic information

5d. Relevant past interventions with outcomes

6. Describe significant physical examination (PE) and important clinical findings

7. Historical and current information from this episode of care organized as a timeline

8a. Diagnostic testing (such as PE, laboratory testing, imaging, surveys)

8b. Diagnostic challenges (such as access to testing, financial, or cultural)

8c. Diagnosis (including other diagnoses considered)

8d. Prognosis (such as staging in oncology) where applicable

9a. Types of therapeutic intervention (such as pharmacologic, surgical, preventive, self-care)

9b. Administration of therapeutic intervention (such as dosage, strength, duration)

9c. Changes in therapeutic intervention (with rationale)

10a. Clinician and patient-assessed outcomes (if available)

10b. Important follow-up diagnostic and other test results

10c. Intervention adherence and tolerability (How was this assessed?)

10d. Adverse and unanticipated events

11a. A scientific discussion of the strengths AND limitations associated with this case report

11b. Discussion of the relevant medical literature with references

11c. The scientific rationale for any conclusions (including assessment of possible causes)

11d. The primary “take-away” lessons of this case report (without references) in a one paragraph conclusion

12. The patient should share their perspective in one to two paragraphs on the treatment(s) they received

13. Did the patient give informed consent? Please provide if requested

## Discussion

### Indication/practical consideration in pediatrics

Despite achieving complete cytologic remission, patients can still harbor minimal residual disease (MRD) that will eventually lead to relapse. Continuation therapy is therefore imperative to prevent relapse, especially in high-risk MRD-positive patients. TKI continuation therapy post HSCT is specifically indicated for patients with chromosomal alterations that result in fusion proteins with constitutively active tyrosine kinase activity [8, 11]. Continuation therapy can be administered as a prophylactic regimen if MRD is negative post-HSCT, or as a preemptive regimen if MRD is positive post-HSCT [22]. MRD is measured by using RT-PCR to quantify the level of the fusion protein transcript [22].

**Fig. 2:**
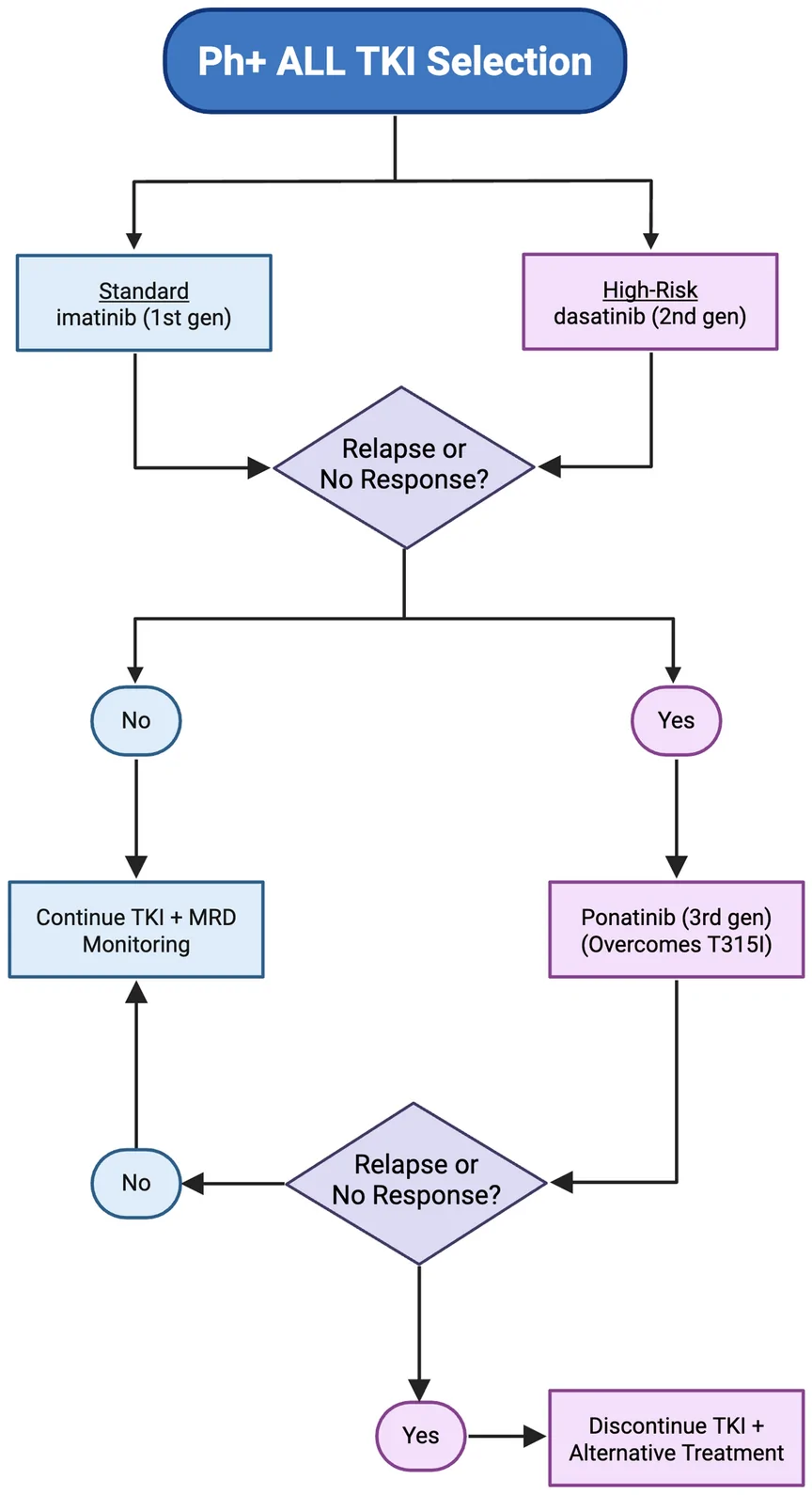
Post-HSCT TKI selection for Ph+ ALL. Created in BioRender. Basson, M. (2026) https://BioRender.com/fl53rjy

Patients with Philadelphia chromosome-positive acute lymphoblastic leukemia (Ph+ALL) have a translocation between chromosomes 9 and 22, producing the BCR-ABL fusion protein [11]. The BCR portion contains an oligomerization domain that brings the ABL kinase domains close together, and this proximity promotes autophosphorylation and constitutive activation, leading to increased cell proliferation and inhibition of apoptosis [23]. Imatinib was the first drug developed to target the BCR-ABL fusion protein. It binds to the ATP-binding site and prevents its subsequent activation and downstream signaling. Second-generation TKIs are often administered to patients with relapsed/refractory Ph+ ALL, especially when these patients are imatinib-resistant [9, 20]. Mutations in the ABL kinase domain can confer resistance to imatinib by preventing the kinase from taking on the closed, inactive conformation to which imatinib binds [24]. Dasatinib, a second-generation TKI, overcomes these mutations by binding to either the active or inactive conformations of BCR-ABL [23]. However, the T315I mutation causes resistance to imatinib and all second-generation TKIs by changing the conformation of the ATP-binding site where TKIs bind [25]. The third-generation TKI ponatinib can overcome this mutation due to its structure-based drug design that allows it to bind to both the wild-type and T315I forms of BCR-ABL with high affinity [25]. The variety of TKIs targeting the BCR-ABL fusion protein emphasizes the need to understand a patient’s disease status and molecular profile in order to choose the most appropriate TKI for post-HSCT continuation therapy (Fig. 2) (Table 3).

**Table 3:** Molecular alterations and corresponding TKIs

Acute leukemia subtype	Mutation/chromosomal alteration	TKI
Ph+ ALL	BCR-ABL fusion kinase	imatinib, dasatinib, nilotinib
	BCR-ABL T315I mutation	ponatinib, olverembatinib
Ph-like ALL	PDGFRB rearrangements	imatinib, dasatinib
	LYN rearrangements	dasatinib
	NTRK rearrangements	larotrectinib
	EPOR rearrangements	ruxolitinib
AML	FLT3-ITD mutation	sorafenib, gilteritinib

Initiating therapy with imatinib allows second and third-generation agents to be preserved for patients who develop resistance or have an inadequate response to imatinib. More recently, later-generation TKIs are selected upfront based on improved survival outcomes compared to imatinib, particularly for high-risk patients with persistent MRD, high leukemic burden, and poor prognosis [26]. CNS involvement is a severe complication of Ph+ ALL and typically leads to poor outcomes. Nilotinib, a second-generation TKI, was shown to be an effective post-HSCT continuation therapy in a pediatric case of CNS relapse [15]. Although nilotinib has a high protein binding affinity, cerebrospinal fluid has a low protein concentration, so there is more free and active nilotinib in the CNS, allowing for sustained MRD negativity and increased TKI continuation efficacy [15]. This observation suggests that drug pharmacology is another important factor to consider when selecting which TKI should be administered.

### Effect of gene mutation and mechanism of resistance

TKI continuation therapy post-HSCT is indicated in patients with chromosomal alterations producing fusion proteins with constitutive tyrosine kinase activity. Ph+ ALL has a well-defined target, the BCR-ABL fusion kinase. In contrast, Ph-like ALL is a form of B-cell ALL that lacks the BCR-ABL fusion protein. Instead, Ph-like ALL contains chromosomal alterations involving the same kinase family and therefore can be targeted by the same TKIs. For example, PDGFRB rearrangements can result in constitutively active fusion kinases, causing increased proliferation and migration of mesenchymal cells [27]. TKIs like imatinib and dasatinib can bind to the ATP-binding sites of PDGFR kinases and block these signaling pathways [11, 16, 27]. Other fusion kinases are caused by LYN rearrangements, which lead to increased B-cell proliferation and can be inhibited by TKIs like dasatinib because LYN is a member of the ABL/Src tyrosine kinase family [10].

Other forms of Ph-like ALL contain chromosomal alterations with alternative kinases, requiring TKIs that specifically target the corresponding kinase family or downstream signaling pathway. NTRK rearrangements can lead to increased proliferation and CNS infiltration, and can be targeted by larotrectinib, a TRK inhibitor that binds to the ATP-binding site and blocks downstream signaling pathways [17, 28]. Furthermore, the EPOR gene itself does not code for a tyrosine kinase receptor, but EPOR rearrangements can cause constitutive activation of JAK/STAT signaling pathways. This can be targeted by ruxolitinib, which competitively binds to the ATP-binding sites of the JAK1 and JAK2 kinases to inhibit downstream signaling [11, 29]. The diversity of fusion proteins that characterize Ph-like ALL emphasizes the need for more personalized post-HSCT continuation strategies based on a patient’s specific molecular profile (Table 3).

Along with Ph+ and Ph-like ALL, acute myeloid leukemia (AML) can also contain mutations leading to constitutively active kinase activity. The FLT3-internal tandem duplication (FLT3-ITD) mutation, although not a fusion protein, leads to constitutive activation of the FLT3 kinase, resulting in increased anti-apoptotic signaling and repressed myeloid differentiation [30]. The FLT3-ITD mutant receptor can be targeted by a multitargeted kinase inhibitor such as sorafenib or by a FLT3 specific inhibitor like gilteritinib, which binds to the ATP-binding site and prevents the autophosphorylation and activation of the FLT3-ITD receptor [8, 19, 30, 31]. With its increased potency and specificity, gilteritinib has been shown to be clinically effective as a post-HSCT continuation therapy in pediatric cases of relapsed FLT3-ITD-positive AML [19].

Due to selective pressure, prolonged TKI continuation therapy may lead to increased cellular resistance mechanisms and further mutations conferring resistance, such as T315I in the BCR-ABL fusion protein. In such instances, switching to a TKI that can overcome the mutation has variable results. In a study containing eight pediatric patients with imatinib-resistant Ph+ ALL, all were treated with a second-generation TKI, but five patients developed the T315I mutation after 4 months [9]. Although these five patients were switched to ponatinib, a third-generation TKI that can overcome this mutation, four of the patients died from relapse, and only one survived until the last follow-up [9]. These findings suggest that prolonged TKI exposure may create selective pressure that favors the expansion of resistant clones, leading to refractory disease that can be lethal for heavily pre-treated patients. In a similar cohort of adult patients with Ph+ ALL previously treated with imatinib and/or dasatinib, the median time of relapse post-HSCT with the T315I mutation was 10.8 months [32]. Of note, all of these patients achieved complete molecular remission post-transplant, indicating that regular MRD monitoring and mutational analysis is critical throughout the duration of continuation therapy to allow for early relapse detection and intervention. Furthermore, sequential TKI treatment increases the risk of compound mutations, which is defined as the presence of two or more mutations within the same BCR-ABL allele, such as E255V/T315I [33]. These mutations can cause resistance even to ponatinib, indicating the need to develop therapeutic strategies that minimize mutational escape [33]. Although not yet studied in pediatric cases of Ph+ ALL, asciminib may be beneficial in this context because it is an allosteric BCR-ABL inhibitor that does not bind to the ATP-binding site unlike traditional TKIs [34].

### Dosage and duration

Based on current studies and reports, pediatric dosage and duration of tyrosine kinase inhibitors, especially as a post-HSCT continuation therapy, are not well-defined and require further testing to establish standard protocols.

Dosage of TKIs is highly variable across studies and dependent on the specific TKI. For Ph+ ALL, imatinib is typically administered between 150–300 mg/m^2^, and dosage adjustment can range from 100–600 mg/m^2^ [9, 13, 16, 21]. The second-generation TKI dasatinib is given at 40–60 mg/m^2^ with dose adjustment ranging from 20–70 mg/m^2^ [9, 10, 13, 14, 20]. Pediatric dosing of TKI is often calculated based on the adult dose, such as nilotinib, which is given to adults at 400 mg but is reduced to 150 mg for children [15]. Dosage of ponatinib and olverembatinib, both third-generation TKIs, is also extrapolated from adult data. In a study administering ponatinib as a post-HSCT continuation therapy, the dose was 15 mg if the child’s body weight was less than 20 kg, 30 mg if the weight was greater than 20 kg, or 45 mg if the weight was similar to that of adults [22]. In a different study administering olverembatinib to pediatric patients with relapsed Ph+ ALL, the post-HSCT dose was started at 20 mg for two patients with body weight at 20 and 29 kg, and at 40 mg for a patient weighing 58 kg [12]. Olverembatinib dose was reduced to 10 mg based on tolerability and adverse reactions [12]. For FLT3-ITD+ AML, pediatric dosage of FLT3 kinase inhibitors is not well-established, and treatment is frequently administered at the adult dosage. In one case, administering sorafenib as a post-HSCT continuation therapy, the dose was 400 mg, while the adult dose ranges from 400 to 800 mg [8]. Similarly, in another case, administering gilteritinib as a post-HSCT continuation therapy, the dose was 120 mg, equivalent to the adult dose [19]. Several studies with adult FLT3-ITD+ AML receiving post-HSCT continuation therapies have established the sorafenib dose at 400 mg and the gilteritinib dose at 120 mg [35, 36].

Duration of TKI continuation therapy is similarly variable across studies but less dependent on the specific type of drug. Continuation therapy is typically initiated between 1–3.5 months after HSCT, and treatment can last between 10 months and 3 years [7–22]. In adults with Ph+ ALL, recommendations for continuation duration range from 1–5 years, with one study observing a median duration of 13.7 months across various TKIs [37]. For adult patients with FLT3-ITD+ AML, post-HSCT continuation is recommended for 2–3 years [35]. Quizartinib has also been evaluated in adults with newly diagnosed FLT3-ITD-positive AML. In the QuANTUM-First trial, single-agent quizartinib was continued for up to 3 years following consolidation chemotherapy and/or allogeneic HSCT [38]. Although pediatric data remain limited, these findings provide additional evidence for the evaluation of FLT3-targeted continuation strategies in pediatric AML. In most reported cases with pediatric patients, TKI continuation therapy continues indefinitely until relapse or adverse events occur [7–22]. In one case of TKI continuation with gilteritinib for a pediatric patient with FLT3-ITD+ AML, the patient was in molecular remission during the 3 years of continuation therapy but relapsed only 3 months after discontinuation. The same patient achieved complete remission after restarting gilteritinib continuation [19]. This observation suggests that continuous TKI therapy may be necessary in certain clinical settings to maintain complete remission.

### Side effects and related toxicities

Several side effects have been observed among patients receiving post-transplant TKIs. Post- HSCT imatinib has led to neutropenia, febrile neutropenia, impaired liver function, and skin reactions, leading to dose and drug modification [21]. Despite these side effects, imatinib was still continued for months following transplant in these patients [21]. Survival remained favorable, suggesting that toxicity- related dose adjustments did not indicate discontinuation of therapy [21].

Second-generation TKIs are also associated with distinct side effects. Dasatinib has been observed and noted to be linked to pleural effusion, thrombocytopenia, and neutropenia in leukemia patients [39]. In a pediatric post-transplant case, cardiopulmonary toxicity was observed, leading to the development of bilateral pleural effusions during treatment [13]. This indicates that though second-generation TKIs have been proven to control disease, they can also introduce pulmonary complications that require careful monitoring and treatment modification. Nilotinib was less frequently reported to be associated with pleural toxicity. However, nilotinib carries risks of metabolic abnormalities and cardiac issues, including hyperglycemia, dyslipidemia, and QT interval prolongation [40].

More adverse toxicity has been reported with third-generation TKIs. Ponatinib, frequently indicated for resistant disease and kinase domain mutations, has demonstrated increased vascular and systemic toxicity in larger leukemia cohorts [41]. In a pediatric post-transplant case, ponatinib therapy was discontinued early due to transaminitis and cytopenias, with subsequent relapse illustrating how intolerance may compromise sustained disease control [11]. Mutation-directed TKIs with narrower kinase inhibition may demonstrate improved tolerability in selected settings. Gilteritinib, for example, has been associated with manageable cytopenias and relatively limited cardiac toxicity in FLT3-mutated AML populations and was tolerated during extended post-transplant administration in a pediatric patient without reported drug-specific adverse events [6, 19].

**Fig. 3:**
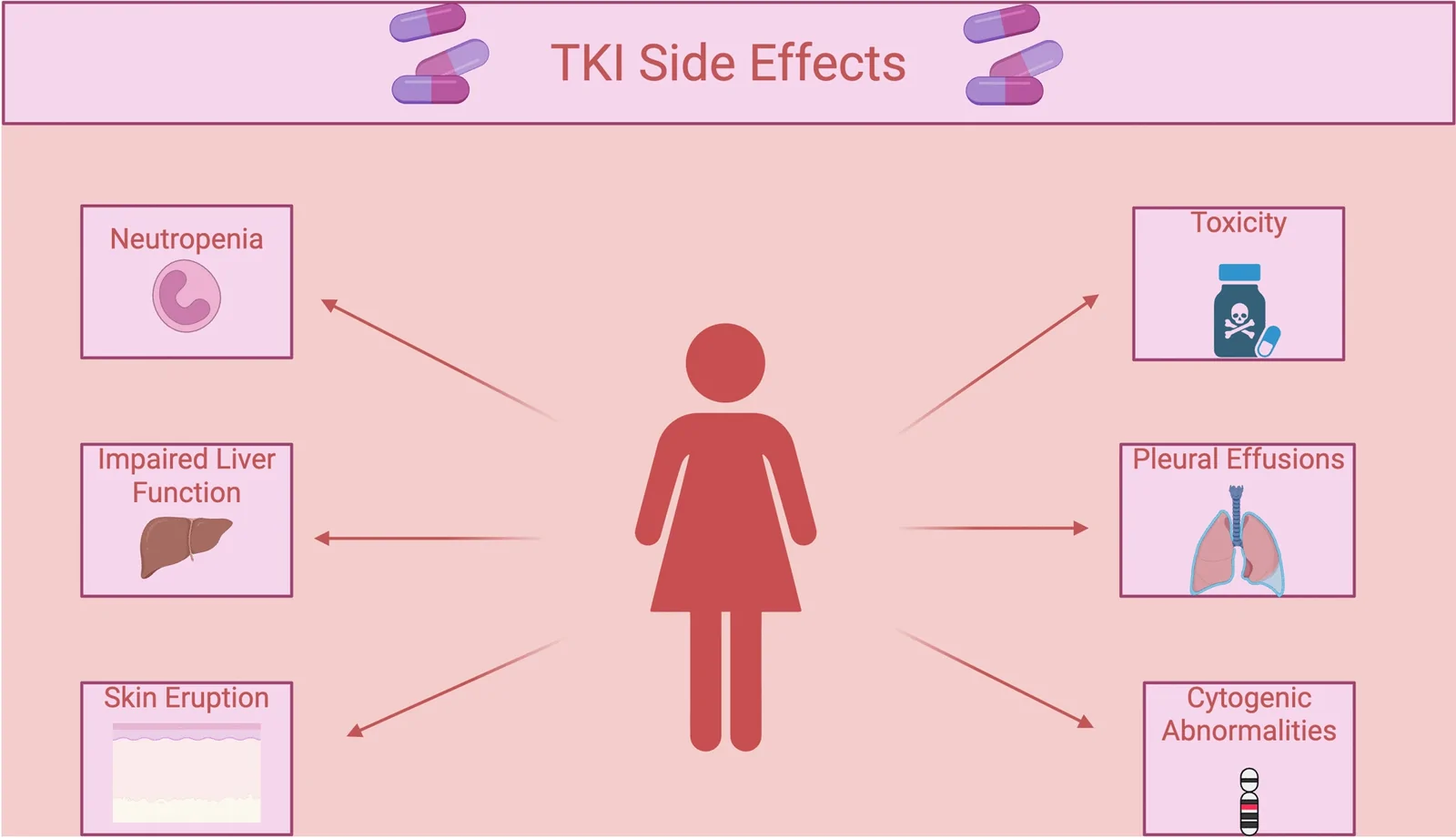
Side effects of post-HSCT TKI continuation therapy. Created in BioRender. Basson, M. (2026) https://BioRender.com/7m9e1dm

Beyond acute hematologic toxicity, additional post-transplant complications have been described. In a pediatric patient receiving sequential imatinib and dasatinib following matched sibling HSCT, clonal cytogenetic abnormalities in Philadelphia chromosome–negative metaphases were observed but resolved after hypomethylating therapy, with long-term hematologic stability maintained [13]. Collectively, these findings demonstrate that post-transplant TKI therapy may be associated with both hematologic and non-hematologic toxicities and reinforce the importance of longitudinal and comprehensive monitoring in pediatric transplant recipients (Fig. 3).

### Impact on transplant and oncologic outcome

In pediatric transplant cohorts, early post-transplant molecular response, particularly BCR-ABL negativity at day +30, was associated with lower relapse rates [9]. In a cohort of pediatric patients undergoing haploidentical HSCT in the imatinib era, 5-year overall survival was 70.0%, 5-year event-free survival was 61.0%, and 3-year cumulative relapse incidence was 22.7% [9]. BCR-ABL transcript positivity at day +30 after transplantation was significantly associated with relapse, underscoring the prognostic importance of early post-transplant molecular disease status [9].

**Fig. 4:**
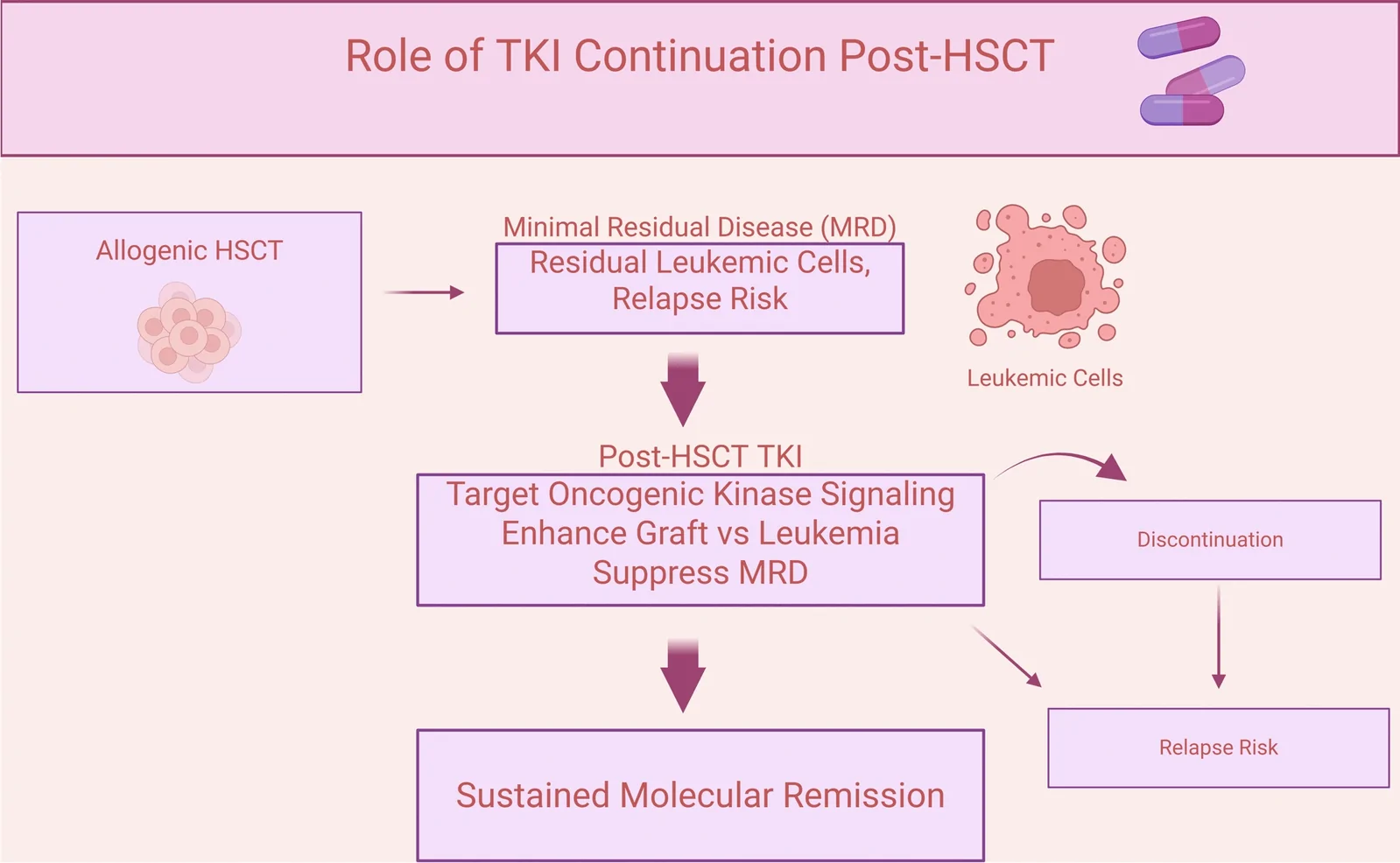
Overview of the role of post-HSCT TKI continuation therapy towards sustaining molecular remission. Created in BioRender. Basson, M. (2026) https://BioRender.com/nl1idal

Post-transplant TKI therapy has also enabled restoration and continuation of molecular remission sufficient to permit repeat transplantation or sustained remission following HSCT. Dasatinib facilitated molecular remission prior to second transplantation in relapsed Ph+ ALL, while prophylactic post-transplant dasatinib was administered for two years, with continued molecular remission after discontinuation [14, 20]. In kinase-fusion–driven ALL, dasatinib administered after HSCT contributed to sustained MRD-negative remission [10]. These reports support the feasibility of post-transplant TKI use in achieving and maintaining molecular disease suppression (Fig. 4).

Durable molecular remission during continued post-transplant targeted therapy has been documented across multiple pediatric cases. Larotrectinib achieved molecular clearance in both bone marrow and central nervous system compartments with sustained remission extending 320 days [17]. Nilotinib produced prolonged remission in post-transplant CNS relapse despite low cerebrospinal fluid drug concentrations [15]. In FLT3-ITD AML, sorafenib and gilteritinib were associated with sustained molecular remission during active therapy, with relapse occurring after discontinuation in one case and remission regained upon re-initiation [19]. Similarly, prolonged remission was observed during post-transplant imatinib therapy in PDGFRB-rearranged ALL [16]. Collectively, these findings demonstrate that molecular disease suppression during active post-transplant TKI exposure is achievable across multiple kinase-driven pediatric leukemias.

However, durable remission was not universal. Early discontinuation of ponatinib due to toxicity was followed by relapse shortly after transplantation [11]. In relapsed Ph+ ALL resistant to multiple agents, ponatinib and immunotherapy did not prevent subsequent relapse after HSCT [18]. Persistent mixed chimerism and cytogenetic abnormalities despite sequential TKI exposure have also been described [13]. These cases illustrate the variability of post-transplant TKI efficacy, particularly in the context of resistant or heavily pretreated disease.

### Clinical implications

Available pediatric data suggest that post-transplant TKI therapy may contribute to the continuation of molecular remission in kinase-driven acute leukemias, particularly when initiated in the setting of measurable residual disease or high relapse risk. Early post-transplant molecular status has demonstrated prognostic significance, with day +30 BCR-ABL positivity associated with relapse, supporting the clinical importance of molecular monitoring and early intervention strategies [9].

Across multiple reports, sustained remission has been observed during ongoing TKI therapy, including in sanctuary sites such as the central nervous system [15, 17]. In contrast, relapse has been reported following early discontinuation due to toxicity or after cessation of prolonged continuation therapy despite prior molecular remission [11, 19]. These observations suggest that continuity and duration of post-transplant TKI exposure may influence disease control, although optimal duration remains undefined in pediatric populations.

Finally, the selection of TKI appears to require mutation-specific consideration. Dasatinib demonstrated activity in BCR-ABL–positive and kinase-fusion–driven leukemias post-transplant [10, 14, 20]. Nilotinib achieved remission in CNS relapse despite low CSF penetration [15]. In FLT3-ITD AML, sorafenib and gilteritinib were used as post-transplant strategies with sustained remission during therapy, and relapse was observed after discontinuation in selected cases [19]. These reports collectively support a mutation-informed, individualized approach to post-transplant TKI therapy rather than uniform continuation protocols.

### Evidence gaps and future research directions

Despite encouraging findings supporting the use of TKIs as post-HSCT continuation therapy in pediatric acute leukemia, substantial evidence gaps remain. Most available studies are limited to small retrospective cohorts and case reports, limiting generalizability and standardized treatment recommendations. There is also substantial variability in leukemia subtype, molecular profile, TKI selection, dosing, duration of therapy, and MRD monitoring strategies. In addition, long-term pediatric safety data remain limited. Published prospective pediatric trials incorporating post-HSCT TKI therapy remain limited to FLT3-ITD AML, including sorafenib in COG AAML1031 and gilteritinib in the SKIPPER trial (Table 4) [41, 42]. Future research should prioritize prospective multicenter studies and collaborative pediatric registries with standardized molecular monitoring, toxicity reporting, and long-term follow-up. Randomized studies comparing TKI agents, duration of continuation, and mutation-directed strategies are also needed to establish evidence-based guidelines for individualized post-transplant management in pediatric acute leukemia.

**Table 4:** Published Pediatric Acute Leukemia Trials Including Post-HSCT TKI Continuation Therapy

Trial	Phase/design	Population	TKI	Post-HSCT TKI component	Key endpoints
Pollard et al., 2022 [42]	Phase 3, non-randomized sorafenib cohort	Children and young adults; de novo high allelic ratio (> 0.4) FLT3-ITD AML	Sorafenib	Sorafenib with chemotherapy, followed by 1 year of single-agent continuation therapy starting 40–100 days after HSCT (or after chemotherapy)	Feasibility; EFS, relapse risk, and DFS
Connor et al., 2026 [43]	Phase 1/2; terminated after phase 1 (enrollment challenges)	6 months to <21 years; relapsed/refractory FLT3-ITD AML	Gilteritinib	Gilteritinib + FLAG, then up to 24 cycles of gilteritinib monotherapy after chemotherapy or after HSCT	Safety; RP2D (2 mg/kg/day for ages ≥2 years); response

Includes published prospective trials enrolling patients younger than 18 years with acute leukemia in which a TKI was administered after allogeneic HSCT. Abbreviations: DFS, disease-free survival; EFS, event-free survival; FLAG, fludarabine, cytarabine, and granulocyte colony-stimulating factor; HSCT, hematopoietic stem cell transplantation; RP2D, recommended phase 2 dose; TKI, tyrosine kinase inhibitor

### Limitations

The review is limited by its retrospective nature, which introduces potential for selection bias. The available pediatric literature on post-transplant TKI use is also limited, with most included studies consisting of case reports and small case series. In addition, the majority of included patients involve pediatric ALL, with limited AML representation. This restricts generalizability and prevents definitive conclusions regarding efficacy or standardized post-transplant continuation strategies in pediatric acute leukemia. Additionally, although a structured screening process was used, article selection and data extraction remain subject to potential human error.

## Conclusion

The use of TKIs as a post-transplant continuation therapy can significantly reduce relapse in pediatric patients with kinase-driven acute leukemias. MRD status and underlying molecular and resistance mutations strongly influence treatment effectiveness. In some cases, treatment interruption and intolerance may lead to relapse; continuous therapy and careful monitoring are essential for sustained remission. Collectively, this emphasizes the need for individualized post-transplant TKI selection. Variability in TKI dosing, duration, and toxicity profiles can also complicate long-term management. This highlights the importance of ongoing molecular surveillance and personalized treatment strategies to optimize disease control and minimize treatment-related side effects and toxicities.

## Supplementary Information

Below is the link to the electronic supplementary material.

Supplementary file1 (DOCX 43 kb)

## Data Availability

Data sharing is not applicable, as no new data was generated or collected for this study. All extracted data were based on previously published data, which are cited within the manuscript.
