# Supplementary material for: The role of tyrosine kinase inhibitors as a post-hematopoietic stem cell transplant continuation therapy for pediatric acute leukemia: a systematic review
Source: Cancer Chemother Pharmacol. 2026 Oct 27;96(1):126. doi: 10.1007/s00280-026-04976-9 (PMC13714093; doi:10.1007/s00280-026-04976-9)
Supplement: Supplementary file 1 [file 280_2026_4976_MOESM1_ESM.docx]

**Supplementary Table 1. Search Query**

| Search strategy | Pubmed | (((("leukemia" [Mesh] OR Leukemia OR Leucocythaemia OR leukemic neoplasm) AND ("Hematopoietic Stem Cell Transplantation"[Mesh] OR hematopoietic stem cell transplant OR Stem cell transplantation OR Transplantation)) AND ("Pediatrics"[Mesh] OR pediatrics OR child OR children OR infant OR infants OR newborn OR neonate)) AND (maintenance therapy OR continuation therapy OR prolonged therapy OR sustained therapy OR long-term therapy OR post-remission therapy OR ongoing therapy OR follow-up treatment OR maintenance treatment OR extended therapy OR chronic therapy)) AND ("Tyrosine Kinase Inhibitors"[Mesh] OR tyrosine kinase inhibitors) |
| --- | --- | --- |
|  | Cochrane | (hematopoietic stem cell transplant OR Stem cell transplantation OR Bone Marrow Transplantation OR Hematopoietic Cell Transplantation) AND (leukemia OR blood cancer OR hematologic malignancy OR haematologic malignancy OR hematopoietic cancer OR malignant blood disorder OR bone marrow cancer OR white blood cell cancer OR acute leukemia OR acute blood cancer OR lymphoblastic leukemia OR lymphoid leukemia OR myeloid leukemia OR myelogenous leukemia OR myeloblastic leukemia OR acute lymphoblastic leukemia OR precursor B-cell leukemia OR precursor T-cell leukemia OR acute myeloid leukemia OR myelogenous acute leukemia OR myeloblast leukemia OR hematologic neoplasm OR hematologic tumor OR malignant hematopoiesis OR clonal hematopoietic disorder) AND (pediatric OR child OR children OR infant OR infants OR newborn OR neonate OR neonates OR adolescent OR teen OR teenager OR youth OR young patient OR minor OR childhood OR pediatric patient OR pediatric population OR juvenile) AND (maintenance therapy OR continuation therapy OR prolonged therapy OR sustained therapy OR long-term therapy OR post-remission therapy OR ongoing therapy OR follow-up treatment OR maintenance treatment OR extended therapy OR chronic therapy) AND (tyrosine kinase inhibitor OR TKI OR tyrosine kinase blockade OR tyrosine kinase antagonists OR kinase inhibitor OR protein kinase inhibitor OR signal transduction inhibitor OR enzyme inhibitor) |
|  | Web of Science | ((((ALL=(leukemia OR blood cancer OR hematopoietic cancer OR bone marrow cancer OR white blood cell cancer OR acute leukemia OR acute blood cancer OR lymphoblastic leukemia OR lymphoid leukemia OR myeloid leukemia OR acute lymphoblastic leukemia OR precursor B-cell leukemia OR precursor T-cell leukemia OR acute myeloid leukemia OR myelogenous acute leukemia)) AND ALL=(hematopoietic stem cell transplant OR Stem cell transplantation OR Bone Marrow Transplantation)) AND ALL=(pediatric OR child OR infants OR newborn OR neonate OR adolescent OR teen OR teenager OR youth OR young patient OR minor OR childhood  OR juvenile)) AND ALL=(maintenance therapy OR continuation therapy OR prolonged therapy OR sustained therapy OR long-term therapy OR post-remission therapy OR ongoing therapy OR follow-up treatment OR maintenance treatment OR extended therapy)) AND ALL=(tyrosine kinase inhibitor OR TKI OR tyrosine kinase blockade OR tyrosine kinase antagonists OR kinase inhibitor OR signal transduction inhibitor OR enzyme inhibitor) |
|  | VHL | (leukemia OR "blood cancer "OR "hematologic malignancy" OR "haematologic malignancy" OR "hematopoietic cancer"OR "malignant blood disorder" OR "bone marrow cancer" OR "white blood cell cancer" OR "acute leukemia" OR "acute blood cancer" OR "lymphoblastic leukemia" OR "lymphoid leukemia" OR "myeloid leukemia" OR "myelogenous leukemia" OR "myeloblastic leukemia" OR "acute lymphoblastic leukemia" OR "precursor B-cell leukemia" OR "precursor T-cell leukemia" OR "acute myeloid leukemia" OR "myelogenous acute leukemia" OR "myeloblast leukemia" OR "hematologic neoplasm" OR "hematologic tumor" OR "malignant hematopoiesis" OR "clonal hematopoietic disorder") AND ("hematopoietic stem cell transplant" OR "Stem cell transplantation" OR "Bone Marrow Transplantation" OR "Hematopoietic Cell Transplantation") AND (pediatric OR child OR children OR infant OR infants OR newborn OR neonate OR neonates OR adolescent OR teen OR teenager OR youth OR "young patient" OR minor OR childhood OR "pediatric patient" OR "pediatric population" OR juvenile) AND ("maintenance therapy" OR "continuation therapy" OR "prolonged therapy" OR "sustained therapy" OR "long-term therapy" OR "post-remission therapy" OR "ongoing therapy" OR "follow-up treatment" OR "maintenance treatment" OR "extended therapy" OR "chronic therapy") AND ("tyrosine kinase inhibitor" OR TKI OR "tyrosine kinase blockade" OR "tyrosine kinase antagonists" OR "kinase inhibitor" OR "protein kinase inhibitor" OR "signal transduction inhibitor" OR "enzyme inhibitor") |
| Date initiated | 10/12/25 | |
| Initial findings | Total= 765   - PubMed: 349 - Cochrane: 139 - VHL: 38 - WOS: 239 | |
| Removed before screening (Duplicates) | Excluded= 87  Included= 678 | |

**Supplementary Table 2. Summary of Outcomes**

| Author, Year | Type of Leukemia | Type of TKI | Dosage and Duration | Indication and Practical Consideration in Pediatrics | Biological Mechanism and Clinical Foundation | Effect of Genetic Mutation | Treatment Side Effects and Related Toxicity | Impact on Transplant Related Outcome | MRD or Prophylatic Usage | Clinical Implications | Mechanism of Resistance |
| --- | --- | --- | --- | --- | --- | --- | --- | --- | --- | --- | --- |
| Byun et al., 2015 | Philadelphia chromosome–positive B-cell precursor ALL | Imatinib | Dose: 213 mg/m² median (105–353) Duration: 4.8 months median (4.2–8.4) | -Used in children with Ph+ ALL undergoing allogeneic HSCT -Evaluated for feasibility and effect of pre- and post-HSCT therapy | Targets BCR-ABL tyrosine kinase created by t(9;22) | Ph+ ALL associated with poorer outcomes vs conventional ALL | Dose reductions needed for: Grade 1 neutropenia Grade 3 febrile neutropenia Grade 1 liver dysfunction Grade 1 skin eruption | HSCT 5-year OS: 80.2% HSCT 5-year EFS: 72.9% Post-HSCT imatinib group: 100% alive without relapse | Post-HSCT imatinib given to patients in CR >6 months | - | - |
| Chen et al., 2017 | Philadelphia chromosome–positive acute lymphoblastic leukemia | Imatinib (primary post-transplant prophylaxis) -Dasatinib and Ponatinib used for imatinib resistance | Started median 69 days post-HSCT Dose 200–300 mg/m²/day Duration: ≥3 months recommended, but individual durations not fully listed | Indication:  Post-transplant prophylaxis to prevent relapse in pediatric Ph+ ALL.  Practical considerations explicitly mentioned:  Imatinib could be started only after hematologic recovery.  Therapy required monitoring for resistance mutations (T315I, etc.). | target BCR-ABL produced by the Philadelphia chromosome translocation | T315I mutation detected in two relapsed patients caused resistance to imatinib.  These mutations prompted switching to ponatinib or other TKIs. | - | 5-year EFS: 86.3%  5-year OS: 92.1%  Relapse occurred in 6/50 patients  Imatinib-resistant mutations (T315I) were associated with relapse | Persistent MRD or rising BCR-ABL levels were associated with relapse.  Two patients who relapsed had T315I mutations, leading to imatinib failure. | Post-HSCT TKI maintenance in pediatric Ph+ ALL results in high survival and low relapse | T315I mutation → confers resistance to imatinib.  Patients with this mutation required switching to ponatinib. |
| Chen et al., 2022 | Philadelphia chromosome–positive acute lymphoblastic leukemia (Ph+ ALL)  All patients carried the T315I BCR-ABL mutation | Ponatinib | Starting dose: 15 mg/day  Dose escalated to: 30 mg/day based on tolerance  Duration: continued for 12 months post-HCT unless toxicity or relapse occurred | Pediatric considerations explicitly mentioned:  Pediatric patients (ages 5–16) tolerated ponatinib well  No grade >1 adverse events were observed in pediatric patients  No dose reduction required in pediatric subgroup | targets T315I mutation, which is resistant to first- and second-generation TKIs | All patients had the T315I mutation, which confers resistance to imatinib, dasatinib, and nilotinib  Ponatinib is specifically chosen because it overcomes the T315I mutation | Grade 1–2 elevated liver enzymes  Grade 1–2 skin rash  Grade 1–2 myelosuppression | No transplant-related mortality reported in relation to ponatinib | MRD monitored via BCR-ABL PCR. Used to guide pre-emptive ponatinib (start therapy when MRD-positive). Rising MRD triggered mutation testing and therapy adjustment. | Ponatinib maintenance reduces relapse in high-risk Ph+ ALL with T315I  Therapy is safe and tolerable, including in pediatric patients  Ponatinib provides an effective post-transplant strategy to overcome T315I resistance | - |
| Dai et al., 2020 | Relapsed B-cell acute lymphoblastic leukemia | Dasatinib | 60 mg/m2 started 8 weeks post transplant and continues for 10 months | Used because the leukemia expressed an NCOR1–LYN fusion, activating LYN kinase, which is sensitive to dasatinib. | NCOR1–LYN fusion results in constitutive activation of the LYN kinase, which is a known dasatinib target | NCOR1–LYN fusion drove leukemogenesis and predicted dasatinib responsiveness.  The patient achieved remission rapidly due to fusion-driven LYN kinase activation, which dasatinib inhibits | - | Patient received haploidentical HSCT.  Post-transplant dasatinib was well tolerated.  The child remained in ongoing remission | - | Dasatinib can be effective for ALL cases driven by rare kinase fusions such as NCOR1–LYN.  Post-transplant dasatinib can be well tolerated in pediatric patients | - |
| Grossman et al., 2004 | Philadelphia chromosome–positive acute lymphoblastic leukemia | Imatinib mesylate | - | - | - | - | Diffuse hypopigmentation of skin and hair occurred while on imatinib.  The hypopigmentation reversed after withdrawal of imatinib | - | - | - | - |
| Li et al., 2023 | Relapsed/Refractory Philadelphia-positive Acute Lymphoblastic Leukemia (Ph+ ALL) | Olverembatinib (HQP1351) | Dose: 30 mg every other day | Patients had failed prior chemotherapy and/or earlier TKIs, and in some cases CAR-T before HSCT. | A third-generation BCR-ABL1 TKI designed to target ABL1 mutations and resistant disease. | - | Cytopenias, Liver enzyme elevations | Maintained remission | - | Patients remained in remission with post-cellular therapy continuation | - |
| Lockhart et al., 2012 | Ph+ ALL | imatinib and dasatinib | imatinib: 230 mg/m2 daily started on day 78 post transplant, increased to 340 mg/m2 4 weeks later, increased to 600 mg/m2 on day 521  dasatinib: 50 mg/m2 twice daily started on day 640, increased to 70 mg/m2 4 months later, 22 months of dasatinib total | - | mixed chimerism and MRD were seen for 5 years after the transplant so TKI therapy was given preemptively to prevent relapse | BCR-ABL+ | bilateral pleural effusions | mixed chimerism and molecular abnormalities did not decrease with imatinib, so patient switched to dasatinib; on dasatinib there was no change in MRD or mixed chimerism but blood counts were normal with no recurrance; cytogenic abnormalities in Ph- metaphases were detected 17 months after TKI therapy was started but marraw examinations were normal | MRD | Ph- clonal cytogenic abnormalities may appear during TKI therapy but can be treated with decitabine | Ph- clonal cytogenic abnormalities may appear after HSCT because donor stem cells are under proliferative stress so genetically unstable clones may emerge |
| Millot et al., 2009 | pre B-cell Ph-positive ALL | imatinib (pre-transplant)  dasatinib (between transplants) | imatinib: 300 mg/m2 daily for 28 days (chemo course 1) then for 14 days per course for 3 courses  dasatinib: 40 mg/m2 twice daily for two months | - | dasatinib targets the BCR-ABL fusion protein | BCR-ABL+ | - | no peripheral blasts on day 11 post dasatinib initiation  no blasts seen after bone marrow aspiration on day 15  0.5x10^9/L neutrophils seen on day 18  50x10^9/L platelets seen on day 26  complete remission after one month of dasatinib (normal peripheral blood counts and marrow)  patient is in complete remission 18 months after second transplant | salvage therapy? | dasatinib can be effective and well-tolerated in heavily pre-treated patients who relapsed after treatment with imatinib | - |
| Reinwald et al., 2014 | BCR-ABL+ ALL | imatinib (pre-transplat)  dasatinib (post-transplant)  nilotinib (post-transplant) | nilotinib 150 mg twice daily, still continues (>650 days) | pediatric dose is 150 mg twice daily while adult dose is 400 mg twice daily | nilotinib has a high protein binding affinity, and CSF has a low protein concentration so there is more free and active nilotinib in the CNS | BCR-ABL+ | - | complete remission in bone marrow and CSF, along with MRD negativity, after 3 months of nilotinib  patient in continuing nilotinib treatment in complete remission (>650 days) | - | nilotinib is active in BCR-ABL+ CNS relapse post-transplant despite low CSF concentration (may be due to low protein concentration in CSF)  2nd gen TKI should be considered for BCR-ABL+ relapse post-transplant | resistance to imatinib could be due to low CNS penetration and increased cellular resistance mechanisms |
| Roldán et al., 2025 | FLT3-ITD+ AML | sorafenib | 200 mg twice daily started on day 112 post-transplant and still continues | - | sorafenib targets the FLT3-ITD mutation | FLT3-ITD mutations cause higher ratess of recurrance and lower post-transplant survival | - | complete remission maintained 12 months after starting sorafenib | prophylactic | sorafenib can be used as a post-transplant maintenance therapy for patients with FLT3-ITD+ AML | - |
| Sakurai et al., 2021 | B-cell precursor ALL (BCP-ALL) | imatinib | 150 mg/m2 daily  pre-transplant for 3 months and post-transplant for 12 months | - | the PDGFRB rearrangement signaling pathway can be targeted by TKIs | BCP-ALL lacks the BCR-ABL gene but these patients will have alterations in other signaling pathways, including PDGFRB rearrangements | - | based on MRD testing every 6 months, patient is in molecular remission during the 24 months post-transplant and 12 months post-TKI | MRD | TKIs can be effective in patients with BCP-ALL that have PDGFRB rearrangements, and Ph+ ALL treatment protocols can be a useful reference | - |
| Schewe et al, 2019 | B-cell ALL | larotrectinib | 100 mg/m2 twice daily from day 55-170  150 mg/m2 twice daily started on day 215 | - | larotrectinib is a selective TRK inhibitor | ETV6-NTRK3+ | - | molecular remission in CNS and bone marrow on day 121; negative MRD in CNS and bone marrow on day 281; patient remain in molecular remission on day 320 | - | larotrectinib can be used to treat ETV6-NTRK3+ B-cell ALL, including patients with CNS infiltration | - |
| Shumock et al., 2023 | patient 3: Ph-like B-ALL patient 4: Ph-like B-ALL patient 5: Ph-like B-ALL | patient 3: dasatinib patient 4: ponatinib and ruxolitinib patient 5: dasatinib (pre and post-transplant) and | patient 3: 2 year goal patient 4: ponatinib for 3 weeks patient 5: about one year after first HSCT and 14 months after second HSCT | - | patient 3: dasatinib targets PDGFRB patient 4: ruxolitinib is a JAK1 kinase inhibitor targeting EPOR::IGH fusion, but ponatinib was used initially to optimize graft-versus-leukemia effects patient 5: PRKCA is a kinase that activates proliferation, apoptosis, and differentiation pathways; PDGFRB fusions produce active tyrosine kinases that can be targeted by TKIs | patient 3: CD74::PDGFRB (out of frame) and RPL11::PDGFRB (in frame); PDGFRB fusion is a constitutively active tyrosine kinase patient 4: EPOR::IGH fusion causes constitutive activation of JAK-STAT signaling pathways patient 5: ETV6::PDGFRB and PRKCA::PDGFRB fusions | patient 4: developed transaminitis and myelosuppression on ponatinib (stopped after 3 weeks) | patient 3: remains on dasatinib and in complete remission at 18 months with negative MRD patient 4: did not tolerate ponatinib and relapsed on day 89; no response to ruxolitinib (in combination with other therapies) patient 5: relapsed (positive MRD) twice on dasatinib post-transplant | MRD | patient 3: dasatinib can be used as a post-HSCT maintenance therapy for Ph-like ALL with a PDGFRB fusion | - |
| Tachibana et al., 2024 | Ph+ALL | dasatinib (pre-transplant)  ponatinib (post-transplant) | one cycle ponatinib between transplants; 4 months after second transplant | - | - | E255K mutation | - | complete remission with negative MRD achieved with after one cyle including ponatinib after first transplant; ponatinib restarted 5 months after second transplant due to positive MRD but relapse occured 9 months post-transplant | MRD | patients with relapsed or refractory Ph+ ALL require personalized treatment plans | TKI therapy not continued after CAR-T cell therapy because patient was already resistant |
| Tasaka et al., 2023 | FLT3-ITD+ AML | sorafenib and gilteritinib | sorafenib started on day 54 after first transplant  gilteritinib started on day 217 (120 mg daily) between transplants, on day 30 after second transplant for 3 years, and on day 40 after third transplant at 40mg then increased to 120 mg on day 27 | - | sorafenib is a multitargeted FTL3 kinase inhibitor  gilteritinib is a selective second generation FLT3 inhibitor | FLT3-ITD+ | - | sorafenib was started after first transplant but relapse occured after 150 days and switched to gilteritinib  molecular remission during 3 years of maintenance therapy with gilteritinib after second transplant, but patient relapsed 3 months after discontinuing gilteritinib, resuming gilteritinib caused blast cells to disappear from peripheral blood  maintenance therapy with gilteritinib after third transplant, complete remission for 3 months (ongoing) | MRD | relapse can occur even after completing long-term maintenance therapy on gilteritinib for patients with relapsed or refractory FLT3-ITD+ AML | since blast cells disappear after resuming gilteritinib, resistance is not due to aquired gilteritinib resistance |
| Watanabe et al., 2013 | Ph+ ALL | imatinib (pre-transplant)  dasatinib (pre and post-transplant) | dasatinib started on day 102 post-transplant at 20 mg/m2 daily, dose was gradually increased to 50 mg/m2 daily and continued for 2 years | - | Dasatinib is a BCR-ABL kinase inhibitor that is 325-fold more potent than imatinib  dasatinib was started after day 100 post transplant at a low dose and gradually increased to avoid adverse effects, it was continued for 2 years with molecular remission because relapse typically occurs within this time frame | BCR-ABL+ | - | patient has been in molecular remission since discontinuing dasatinib | prophylactic | dasatinib may be used as a post-transplant prophylactic to prevent against relapse, but duration and safety needs to be further studied | - |

**Supplementary Table 3. Quality Assessment – STROBE**

|  | 1a | 1b | 2 | 3 | 4 | 5 | 6a | 6b | 7 | 8 | 9 | 10 | 11 | 12a | 12b | 12c | 12b | 12e | 13a | 13b | 13c | 14a | 14b | 14c | 15 | 16a | 16b | 16c | 17 | 18 | 19 | 20 | 21 | 22 |
| --- | --- | --- | --- | --- | --- | --- | --- | --- | --- | --- | --- | --- | --- | --- | --- | --- | --- | --- | --- | --- | --- | --- | --- | --- | --- | --- | --- | --- | --- | --- | --- | --- | --- | --- |
| Byun et al., 2015 [12] |  |  |  |  |  |  |  |  |  |  |  |  |  |  |  |  |  |  |  |  |  |  |  |  |  |  |  |  |  |  |  |  |  |  |
| Chen et al., 2017 [9] |  |  |  |  |  |  |  |  |  |  |  |  |  |  |  |  |  |  |  |  |  |  |  |  |  |  |  |  |  |  |  |  |  |  |
| Chen et al., 2022 [13] |  |  |  |  |  |  |  |  |  |  |  |  |  |  |  |  |  |  |  |  |  |  |  |  |  |  |  |  |  |  |  |  |  |  |

*Green indicated aspects that were complete. Yellow indicates aspects that were unclear if complete. Red indicates aspects that were not complete.

1a. Indicate the study’s design with a commonly used term in the title or the abstract

1b. Provide in the abstract an informative and balanced summary of what was done and what was found

2. Explain the scientific background and rationale for the investigation being reported

3. State specific objectives, including any prespecified hypotheses

4. Present key elements of study design early in the paper

5. Describe the setting, locations, and relevant dates, including periods of recruitment, exposure, follow-up, and data collection

6a. Give the eligibility criteria, and the sources and methods of selection of participants. Describe methods of follow-up

6b. For matched studies, give matching criteria and number of exposed and unexposed

7. Clearly define all outcomes, exposures, predictors, potential confounders, and effect modifiers. Give diagnostic criteria, if applicable

8. For each variable of interest, give sources of data and details of methods of assessment (measurement). Describe comparability of assessment methods if there is more than one group

9. Describe any efforts to address potential sources of bias

10. Explain how the study size was arrived at

11. Explain how quantitative variables were handled in the analyses. If applicable, describe which groupings were chosen and why

12a. Describe all statistical methods, including those used to control for confounding

12b. Describe any methods used to examine subgroups and interactions

12c. Explain how missing data were addressed

12d. If applicable, explain how loss to follow-up was addressed

12e. Describe any sensitivity analyses

13a. Report numbers of individuals at each stage of study—eg numbers potentially eligible, examined for eligibility, confirmed eligible, included in the study, completing follow-up, and analysed

13b. Give reasons for non-participation at each stage

13c. Consider use of a flow diagram

14a. Give characteristics of study participants (eg demographic, clinical, social) and information on exposures and potential confounders

14b. Indicate number of participants with missing data for each variable of interest

14c. Summarise follow-up time (eg, average and total amount)

15. Report numbers of outcome events or summary measures over time

16a. Give unadjusted estimates and, if applicable, confounder-adjusted estimates and their precision (eg, 95% confidence interval). Make clear which confounders were adjusted for and why they were included

16b. Report category boundaries when continuous variables were categorized

16c. If relevant, consider translating estimates of relative risk into absolute risk for a meaningful time period

17. Report other analyses done—eg analyses of subgroups and interactions, and sensitivity analyses

18. Summarise key results with reference to study objectives

19. Discuss limitations of the study, taking into account sources of potential bias or imprecision. Discuss both direction and magnitude of any potential bias

20. Give a cautious overall interpretation of results considering objectives, limitations, multiplicity of analyses, results from similar studies, and other relevant evidence

21. Discuss the generalisability (external validity) of the study results

22. Give the source of funding and the role of the funders for the present study and, if applicable, for the original study on which the present article is based

**Supplementary Table 4. Risk of Bias – ROBINS-I**

|  | D1 | D2 | D3 | D4 | D5 | D6 | Overall Risk of Bias |
| --- | --- | --- | --- | --- | --- | --- | --- |
| Byun et al., 2015 [12] |  |  |  |  |  |  | serious |
| Chen et al., 2017 [9] |  |  |  |  |  |  | low |
| Chen et al., 2022 [13] |  |  |  |  |  |  | moderate |

*Green indicated aspects with low risk. Yellow indicates aspects with moderate risk. Red indicates aspects with serious risk.

D1: Bias due to confounding

D2: Bias in classification of interventions

D3: Bias in selection of participants into the study (or into the analysis)

D4: Bias due to missing data

D5: Bias in measurement of the outcome

D6: Bias in selection of the reported result
